# Food-induced anaphylaxis among a population of adolescents – Report from the BAMSE survey

**DOI:** 10.1186/2045-7022-5-S3-O25

**Published:** 2015-03-30

**Authors:** Mirja Vetander, Jennifer L P Protudjer, Gunnar Lilja, Inger Kull, Marianne van Hage, Anna Bergström, Eva Östblom, Magnus Wickman

**Affiliations:** 1 Sachs’ Children and Youth Hospital, Stockholm, Sweden; 2 Institute of Environmental Medicine, Karolinska Institute, Stockholm, Sweden; 3 Centre for Allergy, Karolinska Institute, Stockholm, Sweden; 4 Department of Clinical Science and Education, Södersjukhuset, Karolinska Institute, Stockholm, Sweden; 5 Clinical Immunology and Allergy Unit, Department of Medicine Solna, Karolinska Institute and University Hospital, Stockholm, Sweden

## Background

The epidemiology and early-life risk factors of food-induced anaphylaxis (“anaphylaxis”) in adolescence are incompletely understood.

## Objective

To study aspects and early-life risk factors of food-induced anaphylaxis amongst adolescents.

## Methods

Parent-reported questionnaire data from 2-3 months, and 1, 2 and 16 years from a large birth cohort were used (N=3153). Immunoglobulin E to 14 common allergens were analysed at 4 (n=2283) and 16 years (n=2510). In a subset of 371 adolescents, 15 additional food allergen extracts or components were analysed at 16 years. Data on dispensed adrenaline autoinjectors and inhaled steroids were extracted from a national register. Severity of food reactions and asthma were defined. The incidence of anaphylaxis was analysed in association with early-life risk factors.

## Results

In the 12 months prior to the study, 8.5% of adolescents reacted to food and 0.8% had anaphylaxis; the annual incidence of the latter was 761/100,000 person years. Only one-third of adolescents when experiencing anaphylaxis had contacted healthcare. Restricting analyses to these adolescents yielded an annual incidence of 240/100,000 person years. In the 24-months prior to the study, adrenaline autoinjectors were dispensed for 67% of those with reported symptoms defined by us as anaphylaxis according to international guidelines. The strongest early-life risk factors for anaphylaxis included sensitisation to foods at 4 years (OR=20.9, 95%CI 6.8-64) and food reactions (OR=17.7, 95%CI 6.91-45.2) between 1-2 years.

## Conclusion

Anaphylaxis in adolescence is more common than previously reported in the literature. Early-life sensitisation and reactions to foods increase anaphylaxis risk in adolescence by more than 15-fold.

